# Epigenomic Indicators of Age in African Americans

**DOI:** 10.4172/2161-1041.1000137

**Published:** 2014-10-01

**Authors:** Jennifer A Smith, Alicia L Zagel, Yan V Sun, Dana C Dolinoy, Lawrence F Bielak, Patricia A Peyser, Stephen T Turner, Thomas H Mosley, Sharon LR Kardia

**Affiliations:** 1 Department of Epidemiology, School of Public Health, University of Michigan, Ann Arbor, Michigan USA; 2 Center for Health Statistics, Washington State Department of Health, Olympia, Washington USA; 3 Department of Epidemiology, Rollins School of Public Health, Emory University, Atlanta, Georgia USA; 4 Department of Environmental Health Sciences, School of Public Health, University of Michigan, Ann Arbor, Michigan, USA; 5 Division of Nephrology and Hypertension, Mayo Clinic, Rochester, Minnesota USA; 6 Department of Medicine and Neurology, University of Mississippi Medical Center, Jackson, Mississippi, USA

**Keywords:** Epigenetics, Epigenomics, Methylation, Age, Aging, African Americans

## Abstract

Age is a well-established risk factor for chronic diseases. However, the cellular and molecular changes associated with aging processes that are related to chronic disease initiation and progression are not well-understood. Thus, there is an increased need to identify new markers of cellular and molecular changes that occur during aging processes. In this study, we use genome-wide DNA methylation from 26,428 CpG sites in 13,877 genes to investigate the relationship between age and epigenetic variation in the peripheral blood cells of 972 African American adults from the Genetic Epidemiology Network of Arteriopathy (GENOA) study (mean age=66.3 years, range=39–95). Age was significantly associated with 7,601 (28.8%) CpG sites after Bonferroni correction for α=0.05 (*p*<1.89×10^−6^). Due to the extraordinarily strong associations between age and many of the CpG sites (>7,000 sites with *p*-values ranging from 10^−6^ to 10^−43^), we investigated how well the DNA methylation markers predict age. We found that 2,095 (7.9%) CpG sites were significant predictors of age after Bonferroni correction. The top five principal components of the 2,095 age-associated CpG sites accounted for 69.3% of the variability in these CpG sites, and they explained 26.8% of the variation in age. The associations between methylation markers and adult age are so ubiquitous and strong that we hypothesize that DNA methylation patterns may be an important measure of cellular aging processes. Given the highly correlated nature of the age-associated epigenome (as evidenced by the principal components analysis), whole pathways may be regulated as a consequence of aging.

## Introduction

Age is a well-established risk factor for chronic diseases [1,2]. However, the cellular and molecular changes associated with aging processes that are related to chronic disease initiation and progression are not well-understood. As the United States transitions into an unprecedented increase in the number of aging adults over the next few decades [3], there is an increased need to identify new markers of cellular and molecular changes that occur during aging processes. These new markers may lead to earlier identification and more effective treatments for chronic disease.

Genetic biomarkers of age include telomere length, gene expression, and DNA methylation patterns. Telomere length decreases with age, and a recent review of 124 cross-sectional studies estimated a mean telomere loss of 24.7 base pairs per year in leukocytes [4]. Some [5,6], but not all [7], cross-sectional and longitudinal studies of telomere length in leukocytes have shown that African Americans have longer telomere lengths than European Americans, after adjusting for age. Telomere loss has also been shown to happen faster in African Americans [5,7]. Since telomere length has also been shown to be associated with chronic disease status, particularly cardiovascular disease [8] and mortality [9], it may serve as an important biomarker for human aging. Gene expression patterns have also recently shown promise as a physical marker of aging in humans. A study by Harries, et al. found that approximately 2% of transcripts genome-wide are robustly associated with age, and that six gene expression probes could be used to build an efficient model to distinguish between younger (<65 years) and older (≥75 years) subjects [10]. To date, little work has been conducted on gene expression patterns and their association with age in African American populations.

Recently, differential DNA methylation patterns that affect gene expression have been shown to be associated with aging [11]. More specifically, age has been found to be associated with methylation status in pathways related to liver development and metabolism [5,12], inflammation, endothelial function, oxidation [13,14], and tumor suppression [15,16]. Since DNA methylation and other epigenetic mechanisms provide a potentially modifiable link between a gene’s expression and a resulting phenotype [17–20], unraveling the relationship between epigenetic mechanisms and cellular aging processes is crucial to understanding the origins of chronic diseases and target organ damage that accompanies aging.

Many prior preliminary studies that have investigated the relationship between DNA methylation and aging processes have either focused on specific genomic regions, such as genes in a single biological pathway [13,14], or have investigated *average* whole-genome DNA methylation [11,21]. Studies of whole-genome methylation have consistently shown an overall decrease in methylation with increased age. Methylome-wide studies conducted in a variety of tissue types and across a wide range of age groups are now emerging [22–27]. These studies have shown significant age-associated changes in DNA methylation at many loci throughout the genome in pediatric (N=398 [23]; N=15 [24]) and adult populations (N=68 [22]; N=63 [23]; N=93 [26]). A few of the age-associated methylation sites have been shown to have a significant overlap between pediatric and adult populations [23,24]; however, the rate of change of DNA methylation with age is estimated to be three- to four-fold faster in pediatric populations [23]. In accordance with the whole-genome methylation studies, the comparison of a newborn and a centenarian genome showed more hypomethylated DNA in the centenarian genome across promoters, exonic, intronic, and intergenic regions, though a greater level of methylation was observed in CpG island promoter regions [27]. Methylome-wide and gene-specific studies have also focused on developing predictive models for age [22,28]. For example, Bocklandt et al. showed that the methylation of three CpG sites is linear with age in adults 18 to 70 years of age, and can predict age with high accuracy (an average of 5.2 years) [22].

Despite the benefits of preliminary studies discussed above, the majority of methylome-wide studies have been conducted in European American samples and/or have consisted of relatively small sample sizes (N<400). In this study, we use genome-wide DNA methylation information from 26,428 individual CpG sites in 13,877 genes to investigate the relationship between age and epigenetic variation in the peripheral blood cells of 972 African American adults from the Genetic Epidemiology Network of Arteriopathy (GENOA) study. We also compare our findings across 4 studies that used the same method for measuring DNA methylation (Illumina Infinium HumanMethylation27 BeadChip) to identify those sites that replicate across studies. Building off of other studies, this work can help to begin identifying the chromosomal regions and pathways involved in the epigenetics of aging.

## Methods

### Sample

The Genetic Epidemiology Network of Arteriopathy (GENOA) study is a community-based study of hypertensive sibships that was designed to investigate the genetics of hypertension and target organ damage in African Americans from Jackson, MS [29]. In the initial phase of the GENOA study (Phase I: 1996–2001), all members of sibships containing ≥2 individuals with essential hypertension clinically diagnosed before age 60 were invited to participate, including both hypertensive and normotensive siblings (N=1,854). In the second phase of the GENOA study (Phase II: 2000–2004), 1,482 participants were successfully re-recruited for a second examination. DNA methylation was measured on 1,008 African American participants using stored blood samples collected during the second (Phase II) examination. The Phase I and II examinations included questionnaires to assess health status, health behaviors, and medical history; physical examination for blood pressure, height, and weight; and fasting blood samples for creatinine, cholesterol, glucose, insulin, and other biochemical measures [30].

### Measurement of DNA methylation

#### Sample preparation and methylation assay

DNA was isolated from peripheral blood leukocytes obtained from stored blood samples, and was bisulfite-converted with the EZ DNA Methylation Gold Kit (Zymo Research, Orange CA). Bisulfite-converted DNA samples were whole-genome amplified, enzymatically fragmented, and purified, then hybridized to Illumina Infinium HumanMethylation27BeadChips, which contain locus-specific DNA oligomers and a set of 56 control probes. The array was then fluorescently stained, scanned using the Illumina BeadXpress reader, and assessed for fluorescence intensities across the methylated and unmethylated bead types at 27,578 CpG sites [31–33]. This work was performed at the Genotyping Core in the Mayo Clinic Advanced Genomics Technology Center (Rochester, MN).

#### Data processing and methylation quantification

At each CpG site, fluorescent signals were measured from the site-specific M (methlyated) and U (unmethylated) bead types. The raw fluorescence data was processed using Illumina BeadStudio. To reduce batch and chip effects, the correlation structure among all 56 control probes was evaluated within channel to identify the most parsimonious subset of probes that explained the maximum amount of batch and chip variation across samples (5 probes in the red channel and 8 probes in the green channel). We adjusted for batch and chip effects by linearly regressing the 13 selected probes onto the intensity signals from the methylated and unmethylated bead types separately across each CpG site.

Before statistical analysis, samples were checked for data quality. Seven samples were excluded from analysis due to poor bisulfite conversion efficiency (intensity <4,000), and an additional 29 samples were excluded due to extreme control probe values (i.e., at least one control probe greater than four standard deviations from its mean value). This resulted in a total sample size of 972.

In this study, we analyzed only autosomal CpG sites. Since our modeling strategy assumes that the error terms for the regression on CpG sites are normally distributed [34], we removed 58 CpG sites from the analysis because they were found to be multimodal based on the Dip Test of unimodality proposed by Hartigan and Hartigan [35] using a cut-off of *p*<0.001 on the signal intensities of the methylated and/or unmethylated bead types. This resulted in 26,428 CpG sites included in our analysis. We next identified the 2,984 CpG sites with non-specific binding probes and 908 CpG sites with polymorphic probes that overlap with single nucleotide polymorphisms (SNPs) reported by Chen et al. [36]. Although these sites were not removed from the analysis, we have interpreted the results from these sites with caution. That is, we acknowledge that the relationship between DNA methylation and age at these sites may be in part influenced by probe characteristics.

Finally, an M-value for each individual *i* at a single CpG site, *k*, was calculated as: M-value_ik_=log_2_[(max(**M**_ik_,0)+1) / (max(**U**_ik_,0)+1)] [37]. Relatively unmethylated M-values were considered to be <−2, methylated M-values were >2, and semi-methylated M-values were between −2 and 2. These M-value cut-offs correspond to β values of 0.2 and 0.8 [37], where β is the ratio of the signal from the methylated probe to the sum of the methylated and unmethylated probes, as follows: β_ik_=max(**M**_ik_,0) / (max(**M**_ik_,0)+ max(**U**_ik_,0)+100). M-values greater than four standard deviations from the mean of each CpG site were removed because these values are discontinuous with the distribution and extend beyond the point where 99.9% of the values are predicted to lie, according to the Empirical Rule [38]. A total of 28,278 outliers were removed from the 26,428 CpG sites included in the analysis. The number of outliers removed ranged from 0 to 34 across all sites (mean=1.07, sd=1.74).

### Statistical analyses

#### Linear mixed effects modeling

We used a linear mixed effects modeling approach to evaluate the cross-sectional associations between DNA methylation and age while accounting for the familial relationships among study participants using the *nlme* package in R [39]. In order to examine the effects of age on DNA methylation, we considered each of the 26,428 individual CpG sites separately as outcomes, with participant age as a covariate in the following model: E_*ijk*_=*β_0_* + *β_1_*Age_*ij*_ + *W_jk_* + *ε_ijk_*, for participant *i* in sibship *j* at CpG site *k*. Age_*ij*_ represents participant age at Phase II exam, *E_ijk_* is the M-value of CpG site *k*, and *W_jk_* is the random effect for each sibship. Thus, in each model, sibship was modeled as a random intercept, and the rest of the effects were modeled as fixed effects. In performing this modeling, four CpG sites exhibited convergence issues and were subsequently removed from the analysis. The Bonferroni method was used to assess experiment-wise statistical significance of the *p*-values (Bonferroni-corrected *p*-value=1.89×10^−6^ for a significance level of α =0.05).

Due to the extraordinarily strong associations between age and many of the CpG sites (>7,000 sites with *p*-values ranging from 10^−6^ to 10^−43^), we wanted to assess the joint effects of CpG sites with age. We first used a set of models to evaluate how well each of the DNA methylation markers predicted age. In these models, age was the outcome and each of the 26,428 CpG sites were predictors, individually, in a linear mixed model: Age_*ijk*_=*β_0_* + *β_1_*E_*ijk*_ + *W_jk_* + *ε_ijk_* for participant *i* in sibship *j* at the *k*^th^ CpG site. We again used the Bonferroni method to assess experiment-wise statistical significance (Bonferroni-corrected *p*-value=1.89×10^−6^).

In order to better understand the joint effects and correlation structure of the large number of CpG sites associated with age, we performed principal component (PC) analysis. We calculated PCs using all 2,095 CpG sites that were significantly associated with age at 1.89×10^−6^. From the scree plot of the PCs, we identified elbow points at 1 PC, 5 PCs, and 10 PCs. Next, we evaluated the bivariate association between age and each of the top five PCs in separate mixed models such that Age_*ij*_=*β_0_* + *β_1_*PC_*ij*_ + *W_j_* + *ε_ij_*. Finally, we evaluated the association between age and the top five PCs combined in a multivariable mixed model such that Age_*ij*_=*β_0_* + *β_1_* PC1_*ij*_ + *β_2_* PC2_*ij*_ + *β_3_* PC3_*ij*_ + *β_4_* PC4_*ij*_ + *β_5_* PC5_*ij*_ + *W_j_* + *ε_ij_*, for participant *i* in sibship *j*. We also constructed a multivariable mixed model using the top 10 PCs. R^2^ values based on likelihood ratio models (R^2^_LR_) were calculated for each model using the R package *lmmfit* [40].

## Results

### Description of data

**Figure 1:**
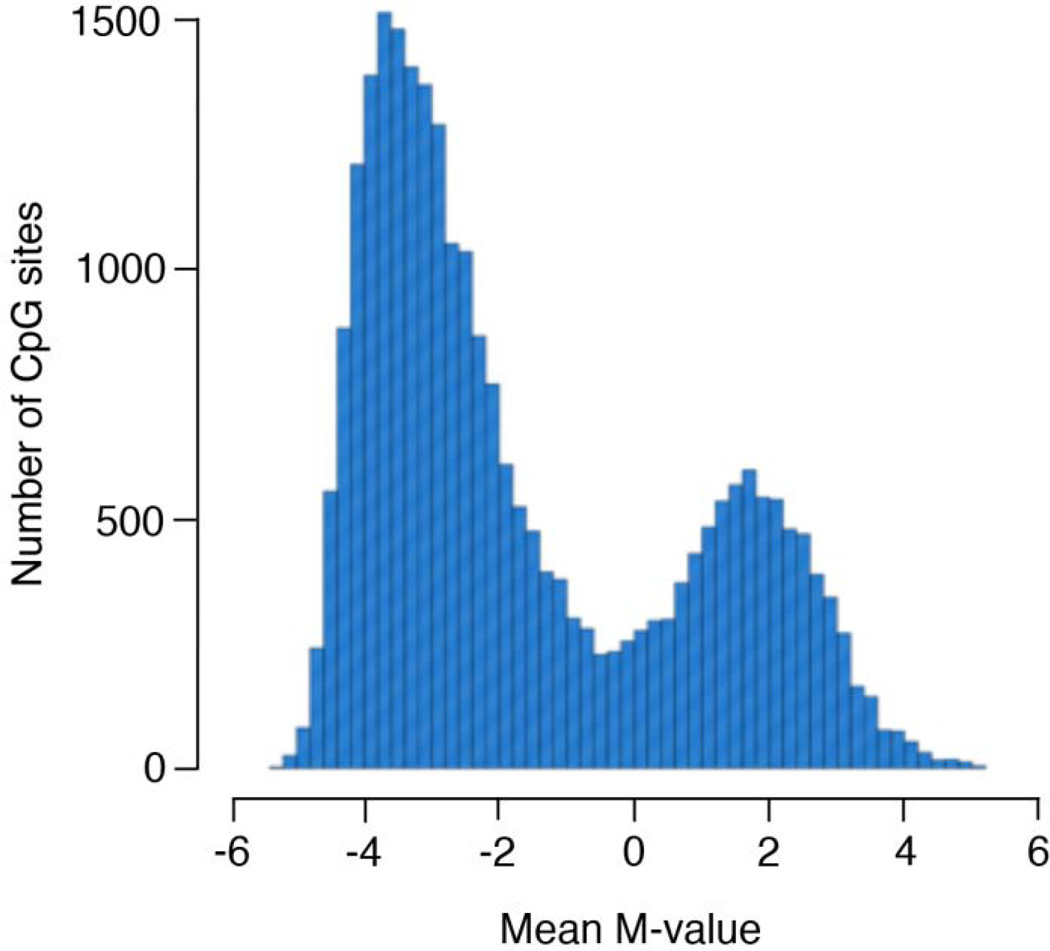
Distribution of mean M-value across 26,428 CpG sites.

After exclusions, this study used phenotype and methylation data from 972 African Americans in 296 sibships across 26,428 CpG sites. The sample was predominantly female (70.7%) and hypertensive (82.5%), with mean age of 66.3 years and mean body mass index (BMI) of 31.2 kg/m^2^. Additional descriptive statistics are presented in Table 1. The mean M-value for each of the 2,428 CpG sites ranged from −5.37 to 5.07 with an average mean M-value across all sites of −1.58 (Figure 1). The majority of the sites (15,221 sites, 57.6%) were unmethylated, with a mean M-value of <−2.

### Associations between age and CpG sites

In modeling age as a predictor of M-value, age was significantly associated with 7,601 (28.8%) CpG sites after Bonferroni correction for α=0.05. Of the sites with statistically significant associations, 671 (8.8%) contained nonspecific binding probes, 159 (2.1%) contained polymorphic probes, and nine sites (0.12%) had both non-specific binding and polymorphic probes as defined by Chen et al. [36]. Adding sex as a covariate into the model did not substantially change the associations between age and the CpG sites (7,410 of the 7,601 associations were still significant after accounting for sex). Table 2 shows the 30 CpG sites that were most strongly predicted by age. A striking finding of this analysis is that age had an inverse association with all but two of the top 30 CpG sites, indicating that increased age is strongly associated with decreased methylation at the majority of the most strongly associated sites.

**Figure 2:**
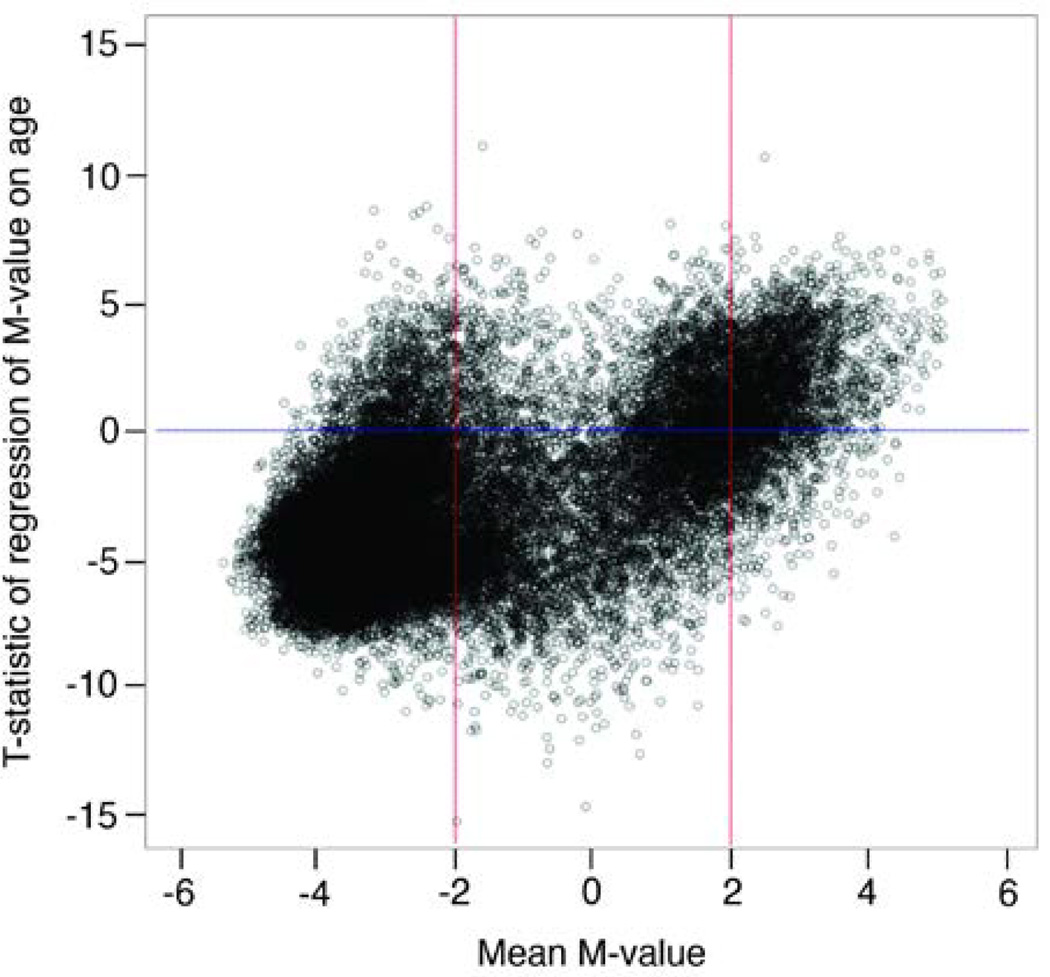
T-statistic distribution of regression of M-value on age vs. mean M-value of corresponding CpG site for 26,428 DNA methylation markers. Red vertical lines at −2 and 2 represent delineation of unmethylated and methylated levels of CpG sites, such that sites having mean M-value <−2 are considered unmethylated, and sites having mean M-value >2 are methylated.

The tendency for age to be inversely associated with CpG site methylation was also observed in the 7,601 CpG site M-values that were significantly predicted by age. Figure 2 shows the relationship between the mean M-value at each of the 26,428 sites and the t-statistic corresponding to the regression coefficient for age. The t-statistic on the y-axis provides two types of information: a) the magnitude of the association with age, and b) the direction of the association with age. For example, a t-statistic of −5.0 represents a *p*-value=5×10^−7^ and indicates that increasing age is associated with decreasing methylation. Of the 7,601 sites statistically significantly associated with age, 7,292 (95.9%) had negative t-statistics, while only 309 (4.1%) had positive t-statistics. Of the 7,292 CpG sites with negative t-statistics, 5,589 sites (76.6%) were unmethylated, 1,675 (23.0%) were semi-methylated, and 28 (0.4%) were methylated. The increased density of negative t-statistics for unmethylated markers (M-values < −2) indicates that they are increasingly *less methylated* with older age. In contrast, of the 309 sites with positive t-statistics, 34 (11.0%) were unmethylated, 106 (34.3%) were semi-methylated, and 169 (54.7%) were methylated. The increased density of positive t-statistics for methylated markers (M-values >+2) indicates that these methylated markers are increasingly *more methylated* with older age. A final feature of the genome-wide results displayed in Figure 2 is that it appears that vast majority of the most significant associations with age (*p*<10^−10^) were in markers that are semimethylated (M-values between −2 and +2).

Given the very large number of highly significant age associations with DNA methylation at CpG sites, we investigated how well the DNA methylation markers could predict age. We examined linear mixed models of CpG site M-values as predictors of age and found 2,095 (7.9%) sites that were significant predictors of age after Bonferroni correction with experiment-wise α=0.05. Supplemental Table 1 shows the 30 CpG sites that had the strongest association with age. Nearly all (2,086, 99.6%) of these sites were also significant in the previously evaluated regression of M-values on age, and had the same direction of effect.

Principal components of the 2,095 age-associated CpG sites were estimated in order to examine the features of the multivariable distribution of significant epigenetic predictors of age (Table 3). The top five principal components accounted for 69.3% of the variability in the 2,095 CpG sites, and the next five principal components accounted for an additional 4.7% (i.e., a total of 74.0%). When each of the top five PCs was used as a predictor of age, each of the first four PCs was significantly associated with age. In a multivariate model, the top five PCs combined explained 26.8% of the variation in age. The linear mixed model containing the top 10 together explained an additional 9.22% (i.e., a total of 36.5%) of the variation in age.

## Discussion

Our findings in GENOA African Americans suggest that age and DNA methylation are very strongly associated at many CpG sites across the genome (28.8% of the CpG sites that we examined). In this study, the associations between the methylation markers and adult age are so ubiquitous and strong that we hypothesize that DNA methylation patterns may be an important measure of cellular aging processes in this population. Given the highly correlated nature of the age-associated epigenome (as evidenced by the principal components analysis), whole pathways may be regulated as a consequence of aging.

Consistent with previous studies in humans and other vertebrates [41–43], we found that the majority of CpG sites (95.9%) tended to be less methylated with increased age (Figure 2). These changes in methylation may contribute to chronic diseases through a variety of mechanisms. For example, it has been found that loss of methylation in CpG dinucleotides over time may transcriptionally activate silenced retrotransposons and lead to genomic instability [44,45]. We also detected a minority of sites (4.1%) that were more methylated with increased age. Increases in methylation at CpG dinucleotides may prevent the binding of transcription factors and potentially suppress gene expression [46]. More investigation of the pathways implicated in these sets of sites may lead to important insights into aging and disease processes. However, replication of these sites would be an important prerequisite to detailed pathway analysis.

Previous research has indicated that DNA methylation is a molecular representation of the cellular memory of environmental experiences. We found that the joint effects of 2,095 CpG sites, represented in the top 10 principal components, were able to explain ~36% of the variation in our GENOA African American adults (mean age=66.3 years; SD=7.6). This indicates that epigenetic markers may be an important link to understanding the genetic and environmental components that contribute to inter-individual differences in the aging process.

Several other studies conducted in a variety of populations have examined the association between age and DNA methylation across the genome using the same Illumina Infinium HumanMethylation27 microarray platform that was used in this study [22–25]. We were able to replicate many of the associations between CpG sites and age that were detected in other studies; however, the extent of replication in GENOA African Americans varied according to the age distribution of the other study population, as well as the tissue type used to measure methylation. Table 4 summarizes the findings from studies that have examined the association between age and DNA methylation and the extent of replication of these findings in GENOA African American adults.

Briefly, we replicated 84.4% of the age-associated CpG sites from a study of saliva samples from 34 monozygotic twin pairs aged 21–55 years conducted by Bocklandt et al. [22] (*p*-value <0.05 in GENOA and the same direction of effect). In a study of whole blood methylation from 398 healthy males aged 3 through 17 years conducted by Alisch et al., we replicated 72.5% of the age-associated CpG sites [23]. Of the sites that we replicated from the Alisch et al. study, the majority (84.6%) were less methylated with increasing age. In order to assess methylation patterns throughout different phases of development, Numata et al. examined methylation in the dorsolateral frontal cortex of the brain in study groups of varying ages (fetal (N=30), childhood, ages 0–10 years (N=15), and beyond childhood, age > 10 years (N=63)) [24]. Despite using a biologically available tissue, we replicated 13%, 49%, and 63% of the frontal cortex age-associated CpG sites in these study groups, respectively. Finally, we replicated 86% of the age-associated CpG sites associated from a study conducted by Teschendorff et al., which examined the association between age and DNA methylation from whole blood samples of postmenopausal women (N=113 ovarian cancer cases and N=148 controls) [25]. Of the sites replicated in GENOA, the majority (69.3%) were less methylated with increasing age.

A variety of factors may have contributed to the differences in findings between the present study and previous studies. Different tissue types display differences in methylation patterns, and there is also a substantial difference between the methylation patterns observed between tissue samples and blood samples [47]. It is not surprising that we replicated a much higher percentage of the age-associated sites from studies that measured methylation in peripheral blood than studies that used tissue samples. Population demographics of the studies may also have contributed to differences in findings. The GENOA population is African American, has an older average age than other populations studied, and is primarily hypertensive. The higher prevalence of hypertension, diabetes, and obesity in this population and/or the higher prevalence of risk factors for these chronic diseases (such as diet, stress, and physical activity) may have led to specific DNA methylation signatures. Since we assessed a cell population of peripheral blood leukocytes that consists largely of neutrophils (40–75%) and lymphocytes (16–48%) [48], we recognize that we may be exploring the aging processes of these cell types which are involved in promoting chronic inflammation, a common correlate of common chronic diseases. Differences in statistical techniques and sample sizes may also have led to differences in the significance levels of age-associated sites, and hence the comparability across studies. However, despite these important differences between studies, we can conclude that there are many CpG sites that are associated with age across a variety of studies, and that our study contributes to a growing body of knowledge that indicate groupings of CpG sites that are important indicators of age and developmental stage across a variety of populations.

Our study does have several limitations. First, as discussed above, the study population is African American, of older age, and primarily hypertensive. Thus, findings may not be entirely generalizable to populations of other ethnic backgrounds, ages, or disease history profiles. However, the GENOA study is a community-based sample that is composed of both hypertensive and normotensive individuals in sibships that have demographics that are similar to other families in the community (age range=39–95 years) [29]. A second limitation is that we do not know the extent to which genetic variation influences epigenetic variation. If there is a substantial influence, then admixture in the African American community from Jackson, MS may affect the results of this study. A third limitation of this study is that we only have cross-sectional measures of methylation and age. Since we do not have longitudinal measures of methylation, we can’t assess how methylation changes with age in individual participants.

This study shows that in this population of GENOA African Americans, many CpG sites are strongly associated with age and predict a substantial amount of variation in age. Future research should include a closer examination of the highly significant markers to determine their molecular physiological role in the aging process. Another avenue of research would be to identify individuals with methylation profiles that are extremely different than their chronological age in order to understand how these markers translate into physiological differences. From a clinical and public health perspective, differences between chronological age and cellular age could be used to identify individuals at greater risk of premature aging and age-related chronic diseases.

## Supplementary Material

Supplementary file

## Figures and Tables

**Table 1:** Baseline characteristics of GENOA participants.

	**Total N**	**Mean (SD)**	**Range**
Age, years	972	66.3 (7.6)	39–95
BMI, kg/m^2^	965	31.2 (6.1)	16.4–55.1
Systolic BP, mm Hg	970	140 (21)	79–221
Diastolic BP, mm Hg	972	78 (11)	45–121
Total cholesterol, mg/dL	972	203.7 (42.1)	73.5–354.5
Triglycerides, mg/dL	963	116.6 (53.8)	37–345
HDL cholesterol, mg/dL	967	57.9 (17.1)	21.7–122.3
LDL cholesterol, mg/dL	972	123.6 (39.7)	24.9–272.1
Glucose, mg/dL	951	108.6 (29.6)	49.5–245
Insulin, mU/mL	953	9.23 (8.25)	0.22–58.29
Serum creatinine, mg/dL	961	0.92 (0.25)	0.42–2.16
	**Total N**	**Count**	**Percent**
Female sex	972	687	70.7%
Ever smoker	909	266	29.3%
Hypertension	972	802	82.5%
Diabetes	972	298	30.7%
Obesity	968	467	48.2%

BMI=body mass index, BP=blood pressure, HDL=high density lipoprotein, LDL=low density lipoprotein.

Obesity is defined as BMI>30 kg/m^2^.

**Table 2:** Top 30 methylation sites most strongly predicted by age.

Outcome	Chr	Gene	Mean (SD) M-value	Probe Type**	N	β(Age)	*p*-value
cg19761273	17	*CSNK1D*	−1.98 (0.3)	0	972	−0.0179	8.45E-43
cg15538427	11	*LOC221091*	−0.11 (0.22)	0	969	−0.0128	3.24E-40
cg01820374	12	*LAG3*	−0.67 (0.31)	0	970	−0.0160	6.23E-33
cg17471102	19	*FUT3*	0.67 (0.29)	0	969	−0.0145	1.64E-31
cg15804973	6	*MAP3K5*	−0.63 (0.34)	0	972	−0.0167	1.14E-30
cg03996822	4	*RASSF6*	−0.21 (0.33)	0	972	−0.0161	2.67E-29
cg25538571	8	*FLJ46365*	−0.67 (0.31)	1	972	−0.0149	7.08E-29
cg00451635	16	*EMP2*	0.62 (0.33)	0	969	−0.0156	2.34E-28
cg19722847	12	*IPO8*	−1.78 (0.32)	0	971	−0.0152	8.15E-28
cg14244577	16	*DDX19B*	−1.7 (0.28)	0	971	−0.0132	8.99E-28
cg08888956	12	*NTS*	0.04 (0.27)	0	972	−0.0128	2.13E-27
cg05442902	22	*P2RXL1*	−1.71 (0.25)	0	971	−0.0115	3.53E-27
cg17034109	1	*CYB561D1*	0.16 (0.25)	0	971	−0.0114	9.10E-27
cg16744741	4	*PRKG2*	−0.46 (0.35)	0	972	−0.0160	5.44E-26
cg15037004	5	*ZNF366*	−0.15 (0.23)	0	970	−0.0107	9.61E-26
cg00431114	20	*C20orf121*	−1.02 (0.27)	0	972	−0.0125	1.60E-25
cg22736354	6	*NHLRC1*	−1.6 (0.39)	0	972	0.0177	2.00E-25
cg00168942	10	*GJD4*	0.05 (0.26)	0	971	−0.0119	4.31E-25
cg07158339	9	*FXN*	−1.19 (0.32)	0	972	−0.0139	5.93E-25
cg04474832	3	*ABHD14A*	−1.72 (0.28)	0	972	−0.0125	6.08E-25
cg27015931	16	*MGC50721*	−2.72 (0.29)	0	971	−0.0127	6.75E-25
cg04662594	8	*EPB49*	−0.81 (0.38)	1	972	−0.0166	2.75E-24
cg03172991	19	*NFIX*	0.53 (0.16)	0	970	−0.0073	3.86E-24
cg08587542	5	*KIAA0141*	−2.42 (0.28)	0	971	−0.0124	4.75E-24
cg05724065	7	*PHKG1*	1.52 (0.28)	0	970	−0.0121	5.03E-24
cg08090640	17	*IFI35*	−1.15 (0.36)	0	971	−0.0160	6.62E-24
cg21232015	12	*CHFR*	2.49 (0.32)	0	970	0.0138	8.36E-24
cg08319238	19	*BCAM*	−1.97 (0.24)	0	970	−0.0105	8.66E-24
cg09706243	11	*POLD4*	−0.97 (0.27)	0	969	−0.0117	1.35E-23
cg03143849	11	*CDKN1C*	−0.22 (0.24)	0	970	−0.0104	1.47E-23

Model: E_*ij*_=β_*0*_ + β_1_Age_*ij*_ + *W_j_*

Probes are designated as polymorphic and/or non-specific binding according to Chen et al. [36].

** 0=Neither polymorphic nor non-specific binding, 1=Polymorphic.

CpG sites listed within this table were not among those with non-specific binding probes.

**Table 3:** Association between top 5 principal components (estimated from 2,095 site M-values significant with age, after Bonferroni correction for α=0.05) and age.

		Univariable Models			Multivariable Model		
PC	% Variation Explained in 2,095 CpG Sites	β	p-value	R^2^_LR_×100	β	p-value	R^2^_LR_×100
1	50.65	−0.12	6.63E-06	12.72	−0.13	5.60E-08	
2	9.53	0.15	0.014	10.34	0.16	3.34E-03	
3	4.52	−0.69	8.84E-14	18.95	−0.72	1.03E-15	
4	2.47	−0.41	4.79E-04	11.39	−0.43	7.45E-05	
5	2.15	0.16	0.21	9.58	0.22	0.22	26.76
6–10	4.68						
Total	74.00						36.54

Model: Age_*ij*_=β_*0*_ + β_*1*_PC_*ij*_ + *W_j_* + ε_*ij*_ (univariable model)

**Table 4:** Comparison of age-associated methylation sites between GENOA and previous studies.

Citation	Sample	Ethnicity	Tissue	Findings	Number of Sites available in GENOA	Number (%) of Sites with Same Direction of Effect and *p*<0.05 in GENOA
Bocklandt et al. [22]	Monozygotic twins (N=34 pairs), ages 21–55 years	Not specified	Saliva	88 CpG sites at *q*-value<0.05 (absolute correlation>0.57)	87	65 (84%)
Alisch et al. [23]	Males ages 3–17 years (N=398)	81% Caucasian, 1% African American, 4% Asian, 14% Other/NA	Peripheral blood cells	2,078 CpG sites at FDR<0.01	2,022	1,465 (73%)
Numata et al. [24]	Fetal (N=30)	40% Caucasian, 60% African American	Dorsolateral frontal cortex	865 at FDR<0.05 (top 99 reported in supplemental material)	96	12 (13%)
	Childhood, ages 0–10 years (N=15)			5,506 at FDR<0.05 (top 99 reported in supplemental material)	99	49 (49%)
	Age>10 to 83 years (N=63)			10,578 at FDR<0.05 (top 99 reported in supplemental material)	99	63 (63%)
Teschendorff et al. [25]	Post-menopausal women, ages 50–84 years (N=113 ovarian cancer cases; N=148 controls)	Not specified	Whole blood	589 at FDR<0.05	583	499 (86%)

Note: All studies measured methylation using the Illumina Infinium HumanMethylation27 BeadChip

## Abbreviations

GENOA: Genetic Epidemiology Network of Arteriopathy
PC: Principal Component
