# Supplementary material for: Epigenomic Indicators of Age in African Americans
Source: Hereditary Genet. Author manuscript; Available in PMC 2016 Jan 21. (PMC4721639; doi:10.4172/2161-1041.1000137)
Supplement: Supplementary file [file NIHMS736146-supplement-Supplementary_file.docx]

Supplemental Table 1. Top 30 methylation sites most strongly associated with age

| CpG site | Chr | Gene | Mean (SD) M-value | Probe Type** | N | β(CpG) | p-value |
| --- | --- | --- | --- | --- | --- | --- | --- |
| cg19761273 | 17 | *CSNK1D* | -1.98 (0.3) | 0 | 972 | -9.36 | 8.51E-36 |
| cg15538427 | 11 | *LOC221091* | -0.11 (0.22) | 0 | 969 | -12.42 | 2.44E-33 |
| cg01820374 | 12 | *LAG3* | -0.67 (0.31) | 0 | 970 | -8.90 | 2.75E-31 |
| cg17471102 | 19 | *FUT3* | 0.67 (0.29) | 0 | 969 | -9.35 | 6.75E-30 |
| cg03996822 | 4 | *RASSF6* | -0.21 (0.33) | 0 | 972 | -7.80 | 1.24E-27 |
| cg22736354 | 6 | *NHLRC1* | -1.6 (0.39) | 0 | 972 | 6.26 | 1.20E-25 |
| cg00451635 | 16 | *EMP2* | 0.62 (0.33) | 0 | 969 | -7.51 | 3.85E-25 |
| cg25538571 | 8 | *FLJ46365* | -0.67 (0.31) | 1 | 972 | -7.71 | 1.39E-24 |
| cg15804973 | 6 | *MAP3K5* | -0.63 (0.34) | 0 | 972 | -7.00 | 4.45E-24 |
| cg08888956 | 12 | *NTS* | 0.04 (0.27) | 0 | 972 | -8.67 | 7.00E-24 |
| cg16744741 | 4 | *PRKG2* | -0.46 (0.35) | 0 | 972 | -6.61 | 2.51E-23 |
| cg14244577 | 16 | *DDX19B* | -1.7 (0.28) | 0 | 971 | -8.24 | 3.92E-23 |
| cg04662594 | 8 | *EPB49* | -0.81 (0.38) | 1 | 972 | -6.04 | 8.22E-22 |
| cg09809672 | 1 | *EDARADD* | -0.35 (0.44) | 0 | 972 | -5.27 | 8.51E-22 |
| cg00431114 | 20 | *C20orf121* | -1.02 (0.27) | 0 | 972 | -8.09 | 1.70E-21 |
| cg17034109 | 1 | *CYB561D1* | 0.16 (0.25) | 0 | 971 | -8.91 | 1.83E-21 |
| cg23124451 | 22 | *CBX7* | 0.37 (0.37) | 0 | 971 | -5.71 | 2.11E-21 |
| cg04474832 | 3 | *ABHD14A* | -1.72 (0.28) | 0 | 972 | -7.75 | 2.17E-21 |
| cg00168942 | 10 | *CX40.1* | 0.05 (0.26) | 0 | 971 | -8.20 | 2.66E-21 |
| cg03172991 | 19 | *NFIX* | 0.53 (0.16) | 0 | 970 | -13.09 | 3.41E-21 |
| cg05724065 | 7 | *PHKG1* | 1.52 (0.28) | 0 | 970 | -7.79 | 7.14E-21 |
| cg09706243 | 11 | *POLD4* | -0.97 (0.27) | 0 | 969 | -8.09 | 9.85E-21 |
| cg05442902 | 22 | *P2RXL1* | -1.71 (0.25) | 0 | 971 | -8.51 | 1.26E-20 |
| cg15297650 | 2 | *DKFZP566N034* | -0.04 (0.29) | 0 | 972 | -7.20 | 1.10E-19 |
| cg10917602 | 16 | *HSD3B7* | 0.42 (0.43) | 0 | 971 | -4.93 | 1.33E-19 |
| cg00308665 | 13 | *HTR2A* | 0.03 (0.3) | 0 | 971 | -7.08 | 1.61E-19 |
| cg17421623 | 3 | *C3orf9* | -1.57 (0.37) | 0 | 971 | -5.67 | 1.78E-19 |
| cg15037004 | 5 | *ZNF366* | -0.15 (0.23) | 0 | 970 | -8.88 | 1.85E-19 |
| cg17324128 | 10 | *RASSF4* | -2.22 (0.28) | 0 | 972 | -7.52 | 2.09E-19 |
| cg20870362 | 9 | *CCIN* | 0.97 (0.23) | 0 | 968 | -9.13 | 2.25E-19 |

Model: Age*_ij_* = *β_0_* + *β_1_* E*_ij_* + *W_j_*

Probes are designated as polymorphic and/or non-specific binding according to Chen et al.^36^

** 0 = Neither, 1 = Polymorphic.

CpG sites listed within this table were not among those with non-specific binding probes.
